# Supplementary material for: Hepatitis E ORF2 Blocks Trophoblast Autophagy to Induce Miscarriage via LC3B Binding Rather than PI3K/Akt/mTOR Pathway Suppression
Source: Microorganisms. 2026 Jan 14;14(1):181. doi: 10.3390/microorganisms14010181 (PMC12843976; doi:10.3390/microorganisms14010181)
Supplement: Supplementary file 1 [file microorganisms-14-00181-s001.zip › supporting information.pdf]

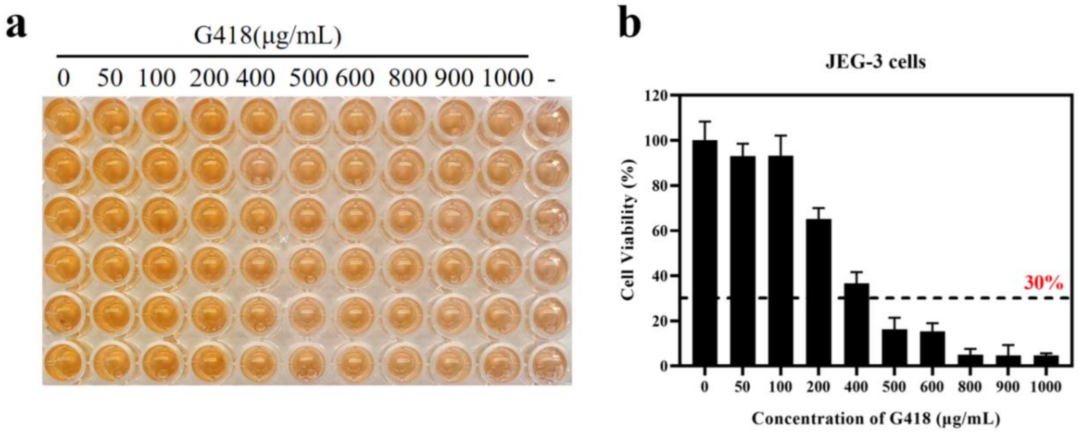


Figure S1. Determination of G418 concentration in screening drug-resistant JEG-3 cells. Legend: (a) The result and (b) quantitative analysis of the G418 cytotoxicity on JEG-3 cells at the concentration of 0-1000 μ g/mL with CCK8 assay. Total cell death is determined by cell activity < 30%, so the concentration of G418 used to screen drug-resistant JEG-3 cells is 500 μ g/mL.

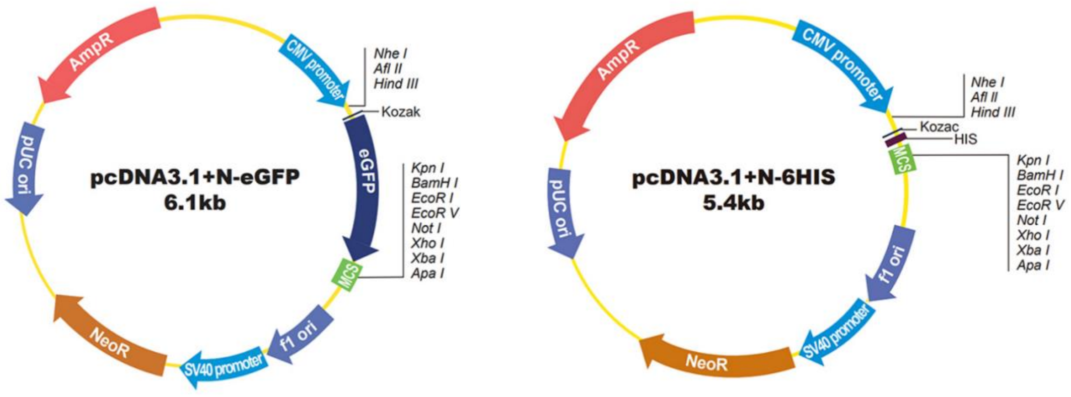


Figure S2. Schematic representation of two plasmid carriers

Table S1. Restriction Enzyme cutting sites and protein sequences of each plasmid

| **Group** | **Plasmid name** | **Restri on Enzyme cutting site** | **Protein sequences** |
|---|---|---|---|
| Control | Flag_pcDNA3. 1(+)-N-eGFP | ApaI and KpnI | DYKDDDDK |
| ORF3 | Flag-ORF3_ pcDNA3.1(+)-N-eGFP | ApaI and KpnI | MNNTFSASPMGSPCALGLFCCCSSCFCL<br>CCPRHRPVSRLAAVVGGAAAVPAVVSG<br>VTGLILSPSPSPIFIQPTPSPLTLTHIPGLEP<br>DPGNQPAPSAHLGATCPSAPPLPRVVDL<br>PQLGLRRDYKDDDDK |
| ORF2 | Flag-ORF2_p cDNA3. 1(+)-N eGFP | ApaI and KpnI | MRTGVVLLLLLVFLPMLPAPPAGQSTGR<br>RRGRRGGGSGGSFWGDRVDSQPFALPY<br>FHPTNPFAADVDSHSGSGARPRQPARPL<br>GTSWRDLSQRPPAASRRRSTPTGAAPLT<br>AVSPAPDTAPVPDVDSRGAILRRQYNLS<br>TSPLTSTVASGTNLVLYAAPLNPLLPLQ<br>DGTNTHIMATEASNYAQYRVVRATIRY<br>RPLVPNAVGGYAISISFWPQTTTTPTSID<br>MNSITSTDVRILVQPGLASELVIPSERLH<br>YRNQGWRSVETSGVAEEEATSGLVMLC<br>IHGSPVNSYTNTPYTGALGLLDFALEVE<br>FRNLTPGNTNTRVSRYTSTARHRLRRGA<br>DGTAELTTTAATRFMKDLHFTGMNGVG<br>EIGRGIALTLFNIADTLLGGLPTELISSAG<br>GQVFYSRPVVSANGEPTVKLYTSVENA<br>QQDKGIAIPHDIDLGDSRVVIQDYDNQH |

| | | | |
|---|---|---|---|
| | | | EQDQPTPSPAPSRPFSVLRANDVLWVSL TAAEYDQTTYGSSTNPMYVSDTVTFVN VATGAQAVARSLDWSKVTLDGRPLTTI QQHSKIFYVLPLRGKLSFWEAGTTKAGY PYNYNPPASDQILIENAADHRVAVSTYP TSLGAGPASISAVGVLAPPSVLAVFEDT VDYPARAHTFDDFRPECRTLGLQGCAF QFTVAELQRLKMKVGKTREFDYKDDD DK |
| mcherry | mcherry_pcDNA3.1(+)-N-6His | EcoRI and XhoI | LGATCPSAPPLPRVVDLPQLGLRRDYKD DDDK |
| mcherry-LC3 | mcherry LC3_pcDNA3.1(+)-N-6His | AflII and EcoRI | MPSEKTFKQRRTFEQRVEDVRLIREQ HPTKIPVIIERYKGEKQLPVLDKTKFL VPDHVNMSELIKIIRRRLQLNANQAF FLLVNGHSMVSVSTPISEVYESEKDE DGFLYMVYASQETFGMKLSVGGGGS |

Table S2. Primer sequence for RT-qPCR amplification

| **Primer Name** | **Primer Sequence (5’ to 3’)** |
|---|---|
| PI3K-F | GAGAAGACCTTCAAGCAG |
| PI3K-R | GAGGCATAGACCATGTACAG |
| Akt-F | ATTGCCGTCTCCGTCTCG |
| Akt-R | CGACGTTGTGGACGTTTACC |
| mTOR-F | TGGCTCCTAGCACCATGAAGAT |
| mTOR-R | GGTGGACAGTGAGGCCAGGAT |
| AMPKA1-F | TTTGCGTGTACGAAGGAAGAAT |
| AMPKA1-R | CTCTGTGGAGTAGCAGTCCCT |
| AMPKA2-F | CTGTAAGCATGGACGGGTTGA |
| AMPKA2-R | AAATCGGCTATCTTGGCATTCA |
| AMPKB1-F | CCATCCTTGATGGAGAGTGC |
| AMPKB1-R | ATCTTGAACAAGGACACGGG |
| TFEB-F | CCAGAAGCGAGAGCTCACAGAT |
| TFEB-R | TGTGATTGTCTTTCTTCTGCCG |
